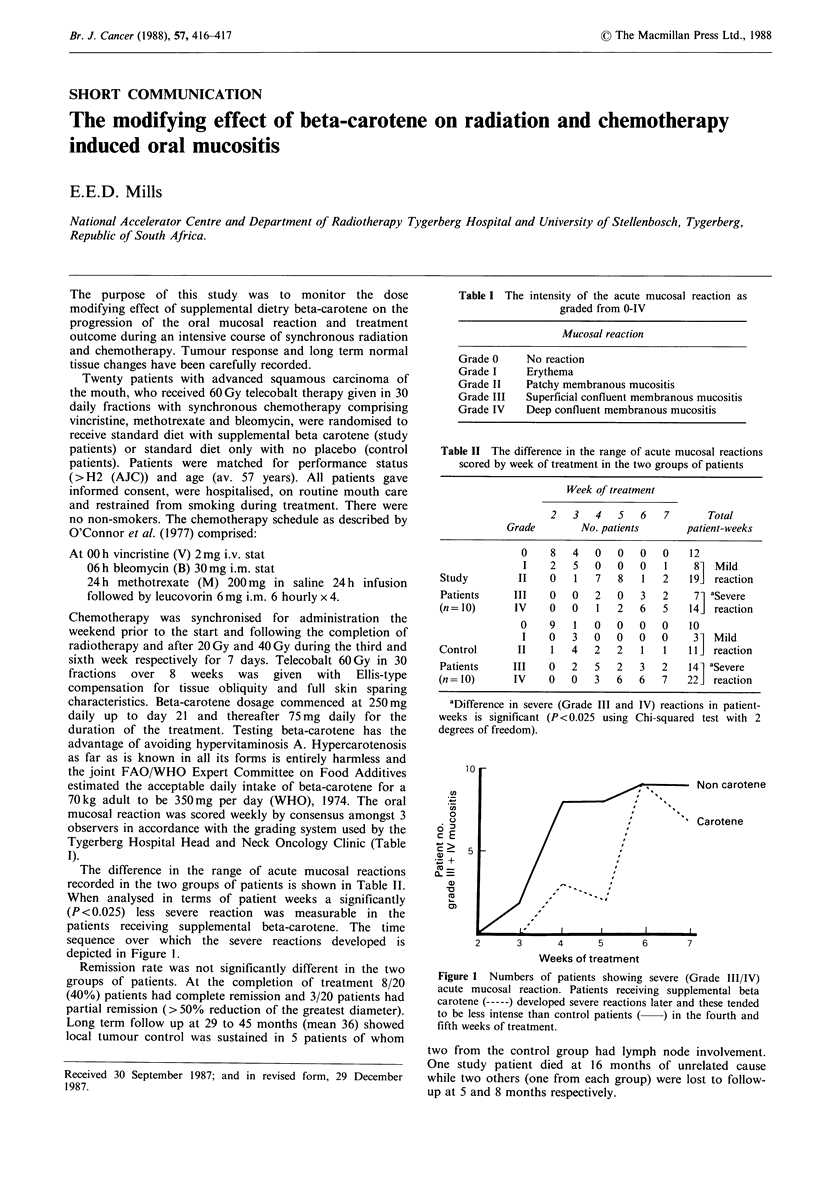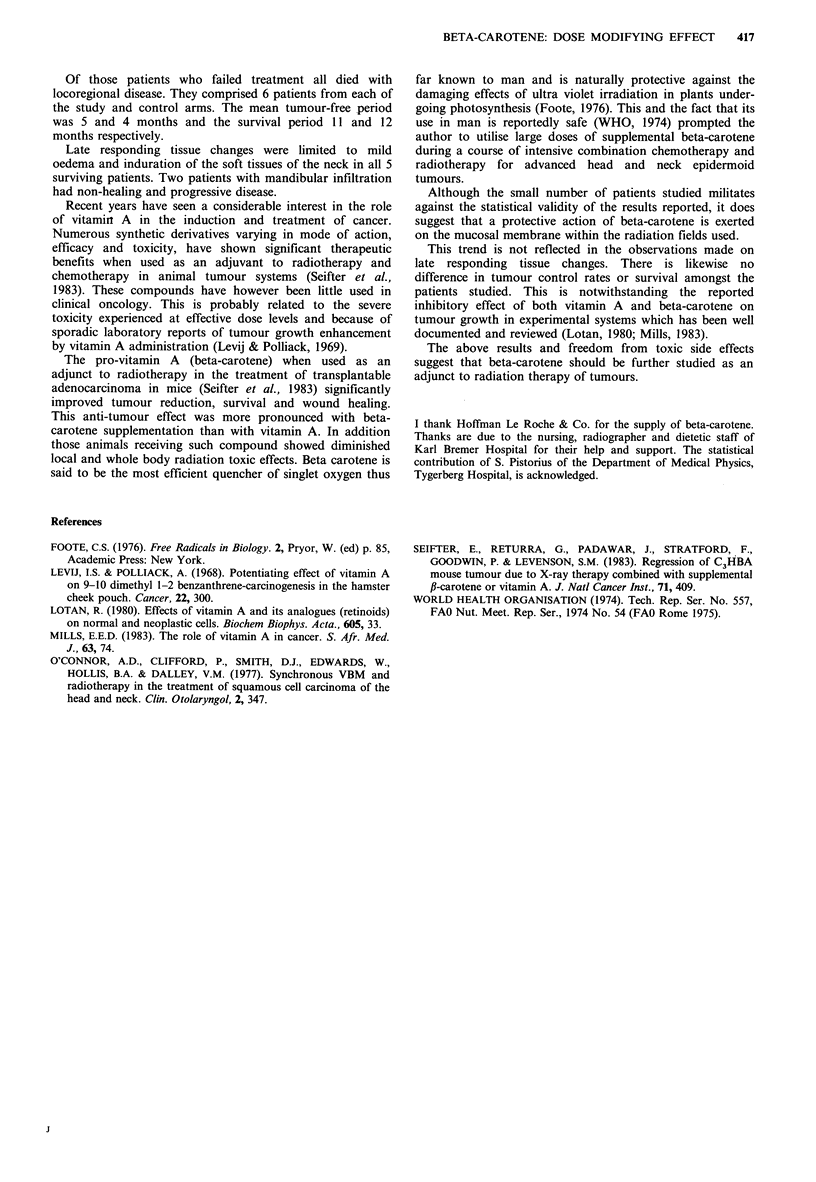# The modifying effect of beta-carotene on radiation and chemotherapy induced oral mucositis.

**DOI:** 10.1038/bjc.1988.94

**Published:** 1988-04

**Authors:** E. E. Mills

**Affiliations:** National Accelerator Centre, Tygerberg Hospital, Republic of South Africa.


*Br. J. Cancer* (1988), **57**, 416–417 © The Macmillan Press Ltd., 1988

SHORT COMMUNICATION

### The modifying effect of beta-carotene on radiation and chemotherapy induced oral mucositis

E.E.D. Mills

*National Accelerator Centre and Department of Radiotherapy Tygerberg Hospital and University of Stellenbosch, Tygerberg, Republic of South Africa.*

The purpose of this study was to monitor the dose modifying effect of supplemental dietry beta-carotene on the progression of the oral mucosal reaction and treatment outcome during an intensive course of synchronous radiation and chemotherapy. Tumour response and long term normal tissue changes have been carefully recorded.

Twenty patients with advanced squamous carcinoma of the mouth, who received 60 Gy telecobalt therapy given in 30 daily fractions with synchronous chemotherapy comprising vincristine, methotrexate and bleomycin, were randomised to receive standard diet with supplemental beta carotene (study patients) or standard diet only with no placebo (control patients). Patients were matched for performance status (>H2 (AJC)) and age (av. 57 years). All patients gave informed consent, were hospitalised, on routine mouth care and restrained from smoking during treatment. There were no non-smokers. The chemotherapy schedule as described by O'Connor *et al.* (1977) comprised:

At 00 h vincristine (V) 2 mg i.v. stat
06 h bleomycin (B) 30 mg i.m. stat
24 h methotrexate (M) 200 mg in saline 24 h infusion followed by leucovorin 6 mg i.m. 6 hourly × 4.

Chemotherapy was synchronised for administration the weekend prior to the start and following the completion of radiotherapy and after 20 Gy and 40 Gy during the third and sixth week respectively for 7 days. Telecobalt 60 Gy in 30 fractions over 8 weeks was given with Ellis-type compensation for tissue obliquity and full skin sparing characteristics. Beta-carotene dosage commenced at 250 mg daily up to day 21 and thereafter 75 mg daily for the duration of the treatment. Testing beta-carotene has the advantage of avoiding hypervitaminosis A. Hypercarotenosis as far as is known in all its forms is entirely harmless and the joint FAO/WHO Expert Committee on Food Additives estimated the acceptable daily intake of beta-carotene for a 70 kg adult to be 350 mg per day (WHO), 1974. The oral mucosal reaction was scored weekly by consensus amongst 3 observers in accordance with the grading system used by the Tygerberg Hospital Head and Neck Oncology Clinic (Table I).

The difference in the range of acute mucosal reactions recorded in the two groups of patients is shown in Table II. When analysed in terms of patient weeks a significantly ($P<0.025$) less severe reaction was measurable in the patients receiving supplemental beta-carotene. The time sequence over which the severe reactions developed is depicted in Figure 1.

Remission rate was not significantly different in the two groups of patients. At the completion of treatment 8/20 (40%) patients had complete remission and 3/20 patients had partial remission (>50% reduction of the greatest diameter). Long term follow up at 29 to 45 months (mean 36) showed local tumour control was sustained in 5 patients of whom two from the control group had lymph node involvement. One study patient died at 16 months of unrelated cause while two others (one from each group) were lost to follow-up at 5 and 8 months respectively.

Received 30 September 1987; and in revised form, 29 December 1987.

**Table I** The intensity of the acute mucosal reaction as graded from 0-IV

| | *Mucosal reaction* |
|---|---|
| Grade 0 | No reaction |
| Grade I | Erythema |
| Grade II | Patchy membranous mucositis |
| Grade III | Superficial confluent membranous mucositis |
| Grade IV | Deep confluent membranous mucositis |

**Table II** The difference in the range of acute mucosal reactions scored by week of treatment in the two groups of patients

| | | *Week of treatment* | | | | | | | |
|---|---|---|---|---|---|---|---|---|---|
| | *Grade* | *2* | *3* | *4* | *5* | *6* | *7* | *Total patient-weeks* | |
| | | *No. patients* | | | | | | | |
| | 0 | 8 | 4 | 0 | 0 | 0 | 0 | 12 | |
| | I | 2 | 5 | 0 | 0 | 0 | 1 | 8 | Mild |
| Study | II | 0 | 1 | 7 | 8 | 1 | 2 | 19 | reaction |
| Patients | III | 0 | 0 | 2 | 0 | 3 | 2 | 7 | [a]Severe |
| ($n=10$) | IV | 0 | 0 | 1 | 2 | 6 | 5 | 14 | reaction |
| | 0 | 9 | 1 | 0 | 0 | 0 | 0 | 10 | |
| | I | 0 | 3 | 0 | 0 | 0 | 0 | 3 | Mild |
| Control | II | 1 | 4 | 2 | 2 | 1 | 1 | 11 | reaction |
| Patients | III | 0 | 2 | 5 | 2 | 3 | 2 | 14 | [a]Severe |
| ($n=10$) | IV | 0 | 0 | 3 | 6 | 6 | 7 | 22 | reaction |

[a]Difference in severe (Grade III and IV) reactions in patient-weeks is significant ($P<0.025$ using Chi-squared test with 2 degrees of freedom).

**Figure 1** Numbers of patients showing severe (Grade III/IV) acute mucosal reaction. Patients receiving supplemental beta carotene (-----) developed severe reactions later and these tended to be less intense than control patients (——) in the fourth and fifth weeks of treatment.

Of those patients who failed treatment all died with locoregional disease. They comprised 6 patients from each of the study and control arms. The mean tumour-free period was 5 and 4 months and the survival period 11 and 12 months respectively.

Late responding tissue changes were limited to mild oedema and induration of the soft tissues of the neck in all 5 surviving patients. Two patients with mandibular infiltration had non-healing and progressive disease.

Recent years have seen a considerable interest in the role of vitamin A in the induction and treatment of cancer. Numerous synthetic derivatives varying in mode of action, efficacy and toxicity, have shown significant therapeutic benefits when used as an adjuvant to radiotherapy and chemotherapy in animal tumour systems (Seifter *et al.*, 1983). These compounds have however been little used in clinical oncology. This is probably related to the severe toxicity experienced at effective dose levels and because of sporadic laboratory reports of tumour growth enhancement by vitamin A administration (Levij & Polliack, 1969).

The pro-vitamin A (beta-carotene) when used as an adjunct to radiotherapy in the treatment of transplantable adenocarcinoma in mice (Seifter *et al.*, 1983) significantly improved tumour reduction, survival and wound healing. This anti-tumour effect was more pronounced with beta-carotene supplementation than with vitamin A. In addition those animals receiving such compound showed diminished local and whole body radiation toxic effects. Beta carotene is said to be the most efficient quencher of singlet oxygen thus far known to man and is naturally protective against the damaging effects of ultra violet irradiation in plants undergoing photosynthesis (Foote, 1976). This and the fact that its use in man is reportedly safe (WHO, 1974) prompted the author to utilise large doses of supplemental beta-carotene during a course of intensive combination chemotherapy and radiotherapy for advanced head and neck epidermoid tumours.

Although the small number of patients studied militates against the statistical validity of the results reported, it does suggest that a protective action of beta-carotene is exerted on the mucosal membrane within the radiation fields used.

This trend is not reflected in the observations made on late responding tissue changes. There is likewise no difference in tumour control rates or survival amongst the patients studied. This is notwithstanding the reported inhibitory effect of both vitamin A and beta-carotene on tumour growth in experimental systems which has been well documented and reviewed (Lotan, 1980; Mills, 1983).

The above results and freedom from toxic side effects suggest that beta-carotene should be further studied as an adjunct to radiation therapy of tumours.

I thank Hoffman Le Roche & Co. for the supply of beta-carotene. Thanks are due to the nursing, radiographer and dietetic staff of Karl Bremer Hospital for their help and support. The statistical contribution of S. Pistorius of the Department of Medical Physics, Tygerberg Hospital, is acknowledged.

### References

FOOTE, C.S. (1976). *Free Radicals in Biology*. **2**, Pryor, W. (ed) p. 85, Academic Press: New York.

LEVIJ, I.S. & POLLIACK, A. (1968). Potentiating effect of vitamin A on 9–10 dimethyl 1–2 benzanthrene-carcinogenesis in the hamster cheek pouch. *Cancer*, **22**, 300.

LOTAN, R. (1980). Effects of vitamin A and its analogues (retinoids) on normal and neoplastic cells. *Biochem Biophys. Acta.*, **605**, 33.

MILLS, E.E.D. (1983). The role of vitamin A in cancer. *S. Afr. Med. J.*, **63**, 74.

O'CONNOR, A.D., CLIFFORD, P., SMITH, D.J., EDWARDS, W., HOLLIS, B.A. & DALLEY, V.M. (1977). Synchronous VBM and radiotherapy in the treatment of squamous cell carcinoma of the head and neck. *Clin. Otolaryngol*, **2**, 347.

SEIFTER, E., RETURRA, G., PADAWAR, J., STRATFORD, F., GOODWIN, P. & LEVENSON, S.M. (1983). Regression of $C_3HBA$ mouse tumour due to X-ray therapy combined with supplemental $\beta$-carotene or vitamin A. *J. Natl Cancer Inst.*, **71**, 409.

WORLD HEALTH ORGANISATION (1974). Tech. Rep. Ser. No. 557, FA0 Nut. Meet. Rep. Ser., 1974 No. 54 (FA0 Rome 1975).